# VTA-NAc glutaminergic projection involves in the regulation of pain and pain-related anxiety

**DOI:** 10.3389/fnmol.2022.1083671

**Published:** 2022-12-07

**Authors:** Mannan Abdul, Hao-Qi Yan, Wei-Nan Zhao, Xiao-Bin Lyu, Zheng Xu, Xiao-Lu Yu, Yi-Hong Gao, Jun-Li Cao

**Affiliations:** ^1^Jiangsu Province Key Laboratory of Anesthesiology, Xuzhou Medical University, Xuzhou, China; ^2^Jiangsu Province Key Laboratory of Anesthesia and Analgesia Application Technology, Xuzhou Medical University, Xuzhou, China; ^3^NMPA Key Laboratory for Research and Evaluation of Narcotic and Psychotropic Drugs, Xuzhou Medical University, Xuzhou, China; ^4^Department of Anesthesiology, The Affiliated Hospital of Xuzhou Medical University, Xuzhou, China

**Keywords:** VTA, NAc, chronic pain, anxiety, glutamate

## Abstract

**Background:**

Besides the established role of dopamine neurons and projections in nociceptive stimuli, the involvement of ventral tegmental area (VTA) glutamatergic projections to nucleus accumbens (NAc) in pain remains unknown. In the present study, we aimed to examine the role of VTA glutamatergic projections to NAc in painful stimuli and its related behavioral changes.

**Methods:**

Unilateral chronic constrictive injury (CCI) of sciatic nerve or intraplantar hind paw injections (i.pl.) of complete Freund’s adjuvant (CFA) were used to develop pathological pain models in wild-type and VGluT2-Cre mice. The involvement of VTA glutamatergic neurons with projections to NAc in CCI-induced pain model was noted by c-Fos labeling and firing rate recordings. Pain response and pain-related behavior changes to the artificial manipulation of the VTA glutamatergic projections to NAc were observed by Hargreaves tests, von Frey tests, open field tests, elevated maze tests, and sucrose preference tests.

**Results:**

Glutamatergic neurons in VTA had efferent inputs to shell area of the NAc. The CCI pain model significantly increased neuronal activity and firing rate in VTA glutamate neurons with projections to NAc. The photoinhibition of these glutamatergic projections relieved CCI-induced neuropathic pain and CFA-induced acute and chronic inflammatory pain. Moreover, pathological neuropathic pain-induced anxiety and less sucrose preference were also relieved by inhibiting the VTA glutamatergic projections to NAc.

**Conclusion:**

Together, glutamatergic inputs from VTA to NAc contribute to chronic neuropathic and inflammatory pain and pain-related anxiety and depressive behaviors, providing a mechanism for developing novel therapeutic methods.

## Introduction

Chronic pain is a hostile sensory and emotional experience related to established or potential tissue damage (Raja et al., 2020). Its negative affective states could also lead to adverse emotional conditions such as anhedonia, fear, anxiety, and depression (Markovic et al., 2021). Nevertheless, the mechanism of chronic pain involves neural complexity among many brain circuits.

Dopamine (DA) release from ventral tegmental area (VTA) neurons is known to provoke pain behavior persistence, stress, anxiety, and addiction (Trainor, 2011). In contrast to the well-studied and established functions of DA neurons, the functional role of VTA glutamate neurons is less studied. It could be due to the relatively small population of glutamate neurons in VTA compared to DA and GABA neurons (Holly and Miczek, 2016; Zhang et al., 2017; Adeniyi et al., 2020). Emerging studies have begun to reveal the reputation of glutamate release from VTA neurons in regulating diverse behavioral repertoire through a complex intra-VTA and long-range neuronal network. The VTA glutamate neurons send projections parallel with VTA DA neurons, such as the nucleus accumbens (NAc) and prefrontal cortex (PFC), and to regions with few VTA dopaminergic inputs, such as lateral habenula (LHb) and ventral pallidum (VP). Some studies reported the role of these glutamate projections in driving aversion and promoting wakefulness, etc. (Zell et al., 2020; Cai and Tong, 2022). Other data suggested that VTA glutamate neurons are more excitable than different types of neurons, as they exhibit increased overall firing to both fear-inducing context memories and increased response to behavioral avoidance, allodynia, and dysregulation of innate defensive behaviors in mice (Root et al., 2018; Barbano et al., 2020; Xia and Kheirbek, 2020). Thus, the VTA glutamatergic neurons may play an important role in pain behavior release.

As mentioned above, NAc is an important down steam nucleus of VTA, which is involved in mediating the reinforcing actions and responses to noxious stimuli. It is also considered that, following peripheral nerve injury, a cell-specific regulation in NAc worsens tactile allodynia (Ren et al., 2016). Besides, deep brain stimulation of the NAc has elicited successful analgesia by forwarding inhibitory projection to the medial thalamus (Strasser et al., 2019; Harris and Peng, 2020).

Even though VTA glutamate and its projections to NAc are being studied in some psychological changes, its role in chronic pain and related emotional behavior remains unclear. Thus, we speculated that VTA-NAc glutamatergic projection regulates pain and pain-related anxiety. In the present study, we investigated the role of VTA glutamatergic projections to NAc in nociceptive response and their related behavioral variations in acute and chronic pain states influenced by the chemogenetic and optogenetic manipulation of VTA-NAc glutamatergic-specific projections.

## Materials and methods

### Animals

Male C57BL/6 J mice (Experimental Animal Center of the Xuzhou Medical University, China) and VGluT2-Cre mice (Jackson Lab, America) were used in the study (Male D1-Cre & D2-Cre mice from Jackson Lab, America, were used in the supplementary experiments). Mice (Maximum, 5 per cage) were housed in a vivarium (22°C–25°C) with free access to food and water under a light/dark cycle of 12 h. All the mice were randomly grouped and subjected to experiments during the light time of the cycle. All the investigators were blinded to experimental conditions during testing. All the experiments were permitted by the Animal Care and Use Committee of Xuzhou Medical University and performed following the Guide for the Care and Use of Laboratory Animals of the National Institutes of Health.

### Adenovirus-associated virus vectors

Adenovirus-associated virus (AAV) vectors purchased from Brain VTA were: rAAV-CaMKIIa-CRE-WPRE-hGH PA AAV2/R; rAAV-Ef1α-DIO-EYFP-WPRE pA; rAAV-Ef1α-DIO-eNpHR3.0-EYFP-WPRE pA; rAAV-EF1α-DIO-hChR2(H134R)-EYFP-WPRE-hGH pA; and rAAV-Ef1α-DIO-hM4D (Gi)-EYFP-WPREs AAV2/R. The virus vectors were of the same batch and title for one complete experiment.

### Stereotaxic surgery and microinjection

Mice were primarily anesthetized with sodium pentobarbital (40 mg/kg, i.p.) and fixed on the small animal stereotaxic apparatus (RWD). After the initial disinfection, the scalp skin was cut to expose the skull’s cranium; 3% hydrogen peroxide was applied to remove the periosteum on the incisional area, and the residual was washed off with the application of normal saline. For the microinjection, the AAV vectors of 200–300 nl volume were injected into the VTA of wild-type mice (AP: 1.05 mm; ML: ±3.20 mm; DV: −4.6 mm; 7° angle) and VTA of VGluT2-Cre mice (AP: 0.4 mm; ML: ±3.10 mm; DV: −4.6 mm) and NAc of wild-type/VGluT2-Cre mice (AP: 1.1 mm; ML: ±0.87 mm; DV: −4.67 mm) by a Hamilton syringe needle, United States (33 gauge) at a rate of 0.1 ml/min, followed by a 10 min pause to minimize backflow. Erythromycin ointment was applied to the wounded site to avoid infection. The ceramic fiber-optic cannulas were implanted bilaterally above the NAc (AP: 1.5 mm; ML: ±1.5 mm; DV: −4.67 mm; 10° angle) and VTA (AP: 1.05 mm; ML: ±3.05 mm; DV: −4.6 mm; 7° angle) of wild-type and VGluT2-Cre mice through the dental cement. Lastly, the mice were placed in sterilized cages with a heating cushion underneath, and these mice were returned to their respective cages on gaining consciousness from anesthesia.

### Pain models

#### Chronic constriction injury of the sciatic nerve

Chronic Constriction Injury of the Sciatic Nerve (CCI) was performed to establish the neuropathic pain model as previously reported (Medeiros et al., 2021). Mice were anesthetized with sodium pentobarbital (40 mg/kg, i.p.). After fur removal and disinfecting of the surgical area, blunt dissection was made to expose the sciatic nerve at the mid-thigh level. Three nonabsorbable 4–0 silks were lightly tied around the sciatic nerve at intervals of 1.0 mm. sham surgery was done without such constrictive ligation as control. After suturing, erythromycin ointment was applied locally on the wound opening. Lastly, the mice were placed in sterilized cages with a heating cushion underneath, and these mice were returned to their respective cages on gaining consciousness from anesthesia.

#### Intraplantar complete Freund’s adjuvant

Complete Freund’s adjuvant (CFA) was injected to establish the inflammatory pain model, as previously reported (Larson et al., 1986). Mice were injected subcutaneously with CFA (10 μl, Beyotime China # P2036) into the plantar surface of the left hind paw plantar (i.pl.) with a 20-gauge micro-injector. Controls were injected with 10 μl saline at the same site of the hind paw.

### Optogenetic stimulation

For optogenetic manipulations, optical fibers in VTA or NAc were connected to a combined laser generator and stimulator (Newdoon, Hangzhou, China), which was used to generate a 594 nm wavelength of the yellow laser, or a 473 nm of a blue laser, with specific output patterns as described in the figures. The three-time periods were selected for “Pre” (baseline observations), “Light” (manipulation through the laser stimulus at 3 h after the baseline readings were observed), and “Post” (4 h post the light stimulus).

### Behavioral tests

#### Paw withdrawal latency

Paw withdrawal latencies (PWLs) were measured using the Hargreaves test (Hargreaves et al., 1988) with the IITC plantar analgesia meter (IITC Life Science). In a quiet environment, these mice were placed in polyethylene cages separately on a glass platform and allowed to accommodate the apparatus for 1–2 h. A radiant heat source beneath the glass was used to stimulate the plantar surface of the hind paw. Before testing, heat intensity was adjusted to produce a baseline of 10–15 s. A cutoff time was set at 25 s to prevent tissue damage. Flinching, flicking, and trembling were considered positive responses. The measurements were triplicated at 10 min intervals, and the mean was calculated as the PWL.

#### 50% paw withdrawal threshold

For the estimation of 50% paw withdrawal threshold (50% PWT), the up- and down-method with von Frey filaments was used. In brief, mice were placed in polyethylene cages separately on an elevated metallic wire mesh platform. Before testing, mice were allowed to acclimatize to the environment for 1–2 h. Testing started with the midrange filament of 0.16 g strength. Subsequent filaments were proceeded according to the up-down method, and 5 consecutive touches were applied at 5 min intervals for rest. The filaments were pressed against the plantar surface and held for 3 s. Positive responses were noted when mice withdrew their hind paw during this time. Finally, 50% PWTs were calculated as described in the previous study (Bonin et al., 2014).

#### Open field test

Open field test (OFT) was conducted with a white plastic open field apparatus (40 cm × 40 cm). This field was artificially divided into a 20 cm × 20 cm center zone and a rest peripheral zone. After sterilization with 75% alcohol, mice were put into the center zone and allowed to travel freely within 10 min. The ANY-maze tracking system recorded the traveling trace, the number of entries into the center zone, and the time spent in the center zone for each mouse.

#### Elevated plus maze

An elevated apparatus with a digital camera was used to perform the EPM test. The maze, 70 cm above the floor, consists of two open arms (30 cm × 5 cm) and two closed arms (30 cm × 5 cm) with 15 cm high opaque walls. When testing, mice were placed in the center area facing an open arm and allowed to travel freely for 5 min. The traveling trace, number of entries into the open arms, and time spent in the open arms for each mouse were recorded by the ANY maze tracking system.

#### Sucrose preference test

Mice were singly housed in two identical leak-resistant bottles containing tap water for 3 days before testing. On testing day, mice first underwent water restriction for 8 h. They were given two identical leak-resistant bottles containing tap water solutions and 1% sucrose solutions. The two bottles of each mouse were exchanged every 6 h. To facilitate the Clozapine-N-oxide (CNO) delivery, the mice were placed under an inverted light/dark cycle, where light onset occurs in the evening. As invasive injections of CNO can manipulate the results, we used the oral route by first dissolving Clozapine-N-oxide (CNO, 5 mg) in 1 ml of 0.9% sterile saline solution and refrigerating the stock solution at 4°C. On test day, we filled the bottles with 10 ml of water +1% sucrose + CNO (1 mg/kg) and 10 ml of water + CNO (1 mg/kg) for the subjected mice, and the control group was placed without the presence of CNO, but with 0.9% sterile saline (1 ml/kg), we noted both the preference in 24 h cycle as reported (Zhan et al., 2019). Following the testing, the percentage of sucrose solution intake was calculated to reflect the sucrose preference.

### Immunohistology and confocal imaging

With deep anesthesia by sodium pentobarbital (40 mg/kg, i.p.), mice were subjected to intracardial perfusion with 40 ml PBS, pH 7.4, and 20 ml 4% PFA. Later, the brain samples were extracted and postfixed in 4% PFA at 4°C for 6–8 h, then kept in 30% sucrose solution for 48 h. Coronal brain sections (40 mm thick) were prepared by a frozen-section microtome (VT1000S, Leica Microsystems). The brain sections with fluorescent protein expression were directly covered, slipped in a mounting medium, and envisioned by a laser scanning confocal microscope (LSM 880, Carl Zeiss). For the desired immunofluorescent staining, all the sections were washed in PBS for 15 min, incubated with an antigen retrieval solution (P0090, Biyuntian) for 5 min, and subsequentially blocked for a nominated period of 1 h with a PBS solution containing 1% BSA and 0.25% Triton X-100. According to the experimental needs of the respected protocols, the sections were incubated with the primary antibodies overnight, including mouse rabbit anti-c-Fos (1:500, 2,250, Cell Signaling Technology). After being washed in PBS for 15 min, the sections were tagged with secondary antibodies for 2 h, like anti-rabbit Alexa-594 (1:200, A21207, Thermo Fisher Scientific). As controls, adjacent sections were incubated without primary antibodies. Lastly, the sections were mounted onto glass slides; images were obtained using a confocal microscope (LSM 880, Carl Zeiss).

### Electrophysiology

The brain was placed after the immediate decapitation at a high concentration of sucrose solution at −4°C, and the brain sheet containing 250 μm thick with VTA and NAc was cut with a vibrating slice. The ingredients of high sugar artificial cerebrospinal fluid are (mm): 254 Sucrose, 1.25 NaH_2_PO_4_, 10 D-Glucose, 24 NaHCO_3_, 3 KCl, 2 CaCl_2_, and 2 MgSO_4_ (PH 7.35, 295–305 MOSM). The brain slice was placed in a 35°C artificial cerebrospinal fluid (ACSF) with 95% O_2_ + 5% CO_2_ mixture for 1 h, and after that, it was placed in 1.25 NaH_2_PO_4_, 10 D-Glucose, 24 NaHCO_3_, 3 KCl, 2 CaCl_2_, and 2 MgSO_4_ (pH 7.35, 295–305 MOSM). After the glass microelectrode (3–5 mΩ) is sealed, the discharge frequency is recorded in the Cell-Attach recording mode. Filtering and collection of the MULTI CLAMP 700B diaphragm amplifier were performed, and light stimulation was given to validate the VTA glutamate neuron downstream toward the NAc for the optogenetic viral vector.

### Statistical analysis

All experiments were replicated in 3–8 mice of each group with the same generation and age, with the data randomly collected and processed. Mice were excluded from data collection and analysis because of the following reasons: (a) because of off-target or poor expression for virus vectors; (b) because of health complications after surgical intervention (e.g., body weight drops 20% in a day) and experimental failures; (c) because of the mortality of mice. No data points were excluded after data acquisition was accomplished. Data were analyzed offline, and experimenters were not blinded to the experimental group during the analyses. All data were expressed as the mean ± SEM. The statistical graph plot and data calculation were performed with GraphPad Prism8. *Two-way ANOVA* with repeated measures followed by *Bonferroni post-tests* was used to compare the differences between the groups with multiple time points of “Pre,” “Light” and “Post.” *Unpaired sample t-tests* were performed in comparing the differences between two groups, *One-Way ANOVA* followed by the *Dunnett’s post-tests* were used in comparing the differences between four groups. Statistical significance was defined as *p* < 0.05. Detailed descriptions can be found in the figure legends. The number of cells expressing c-Fos is compared by using Image-Pro. Plus (Version 6.0.260, Media Cybernetics, United States).

## Results

### CCI surgery increased the excitation level of the glutamatergic neurons projected from VTA to NAc

**Figure 1:**
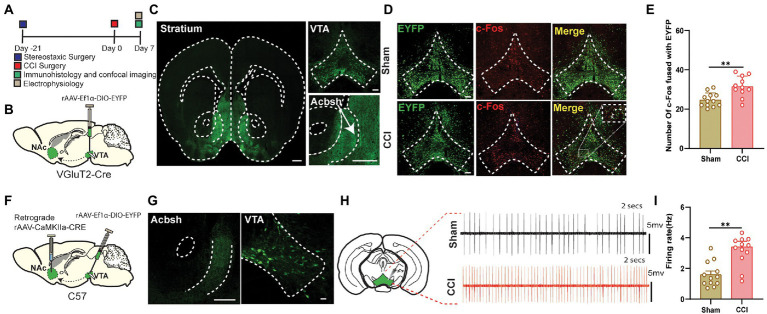
The neuropathic pain can induce hyperactivity in the VTA Glutamate neurons projecting to NAc. **(A)** Experimental timeline. For the immunohistological expression, the DIO-EYFP viral vector was injected in the VTA brain region and VGluT2-Cre mice were given 21 days for the expression of the virus before the CCI surgery. The c-Fos labeling was performed on day 7 after the sham or CCI surgery (unilateral sciatic nerve ligation) in mice. **(B)** Schematic illustration depicting viral constructs and experimental surgery in VGluT2-Cre mice. **(C)** Confocal image of VTA somatic expression of DIO-EYFP (Scale bar = 200 μm) and VTA VGluT2 fibers projecting to NAc (Scale bar = 500 μm and Scale bar = 200 μm); these fibers are highly concentrated in NAc shell. **(D)** Representative confocal images of VTA glutamatergic somatic cell bodies infected by DIO-EYFP and stained with c-Fos of both sham (without nerve ligation) and CCI mice (unilateral sciatic nerve ligation); these images were demarcated as DIO-EYFP only, c-Fos labeled only and merged image of DIO-EYFP infected somatic bodies coupled with c-Fos protein. Scale bar = 200 μm. **(E)** Quantitative data regarding the comparison between c-Fos protein expression coupled with DIO-EYFP infected glutamatergic somatic cell bodies of VTA brain region in the sham group vs. CCI group of the VGluT2-Cre mice (*n* = 12 slices/group from 3, 3 mice; ^**^p < 0.01, unpaired-sample *t*-test). **(F)** Experimental timeline. For the immunohistological expression, retrograde CaMKIIa-CRE viral vector was injected in the NAc brain region, DIO-EYFP viral vector was injected in the VTA brain region, and wild-type mice were given 21 days for the expression of the virus before the CCI surgery. The electrophysiological recordings for the firing rate were done on day 7 after the sham or CCI surgery (unilateral sciatic nerve ligation) in wild-type mice. **(G)** Confocal images showing virus expression in the VTA somatic body and NAc axon terminals in the brain region of wild-type mice infected by the viral vector of CaMKIIa-CRE and DIO-EYFP for the labeling of the glutamatergic projections. Scale bar = 200 μm and Scale bar = 100 μm. **(H)** Traces of firing activity of the glutamatergic somatic bodies of VTA (with axonal projections to NAc) in wild-type mice. **(I)** Quantitative data regarding the firing activity of glutamate neurons in VTA (with axonal projections to NAc) of sham and CCI groups (24 neurons from 6, 6 mice, ^**^p < 0.01, unpaired-sample *t*-test).

We targeted VTA-Glutamatergic neurons and their axons by injecting DIO-EYFP viral vector in the VTA of VGluT2-Cre mice (Figures 1A,B). We found that VTA glutamatergic projection to NAc was localized predominantly in the shell area, and sometimes these fibers are extended into the ventromedial shell and olfactory tubercle (Figure 1C). Then, c-Fos staining was also performed in the VGluT2-Cre mice to determine the neuronal activity in response to painful stimuli. VTA sections were prepared from sham or CCI mice on day 7 post-surgery. The staining results showed that CCI surgery resulted in an increased number of the c-Fos-positive cells in VTA, compared with their sham counterparts (Figures 1D,E). The population of glutamatergic neurons in VTA is reported to be less than other types of neurons like GABA and DA, and we only wanted these glutamatergic projections to be NAc-specific rather than VTA glutamatergic projection to other brain regions in VGluT2-Cre mice. We used the CaMKII tracing method in the wild-type mice because CaMKII was reported to colonize predominantly with the glutamate neurons (Zhu et al., 2014; Basting et al., 2018; Liu et al., 2020; Sheng et al., 2020; Zhang et al., 2020; Li et al., 2022). For this, a retrograde CaMKIIa-CRE viral vector was injected into the NAc, and DIO-EYFP was injected in the VTA of the wild-type mice (Figure 1F). Confocal imaging confirmed that somatic bodies of VTA glutamate neurons had axonal projections to NAc sections were prepared from sham or CCI mice on the day 7 post-surgery (Figure 1G). The firing rates of these VTA glutamatergic somatic bodies were recorded using MULTI CLAMP 700B diaphragm amplifier. We noted a significantly increased firing rate of the neurons in the CCI mice compared to its sham-controlled subjects (Figures 1H,I). The data indicate that the glutamatergic projections from VTA to NAc are involved in the chronic pain mechanism.

### The photoinhibition of the glutamatergic neurons projected from VTA to NAc suppressed CCI-induced pain behavior

**Figure 2:**
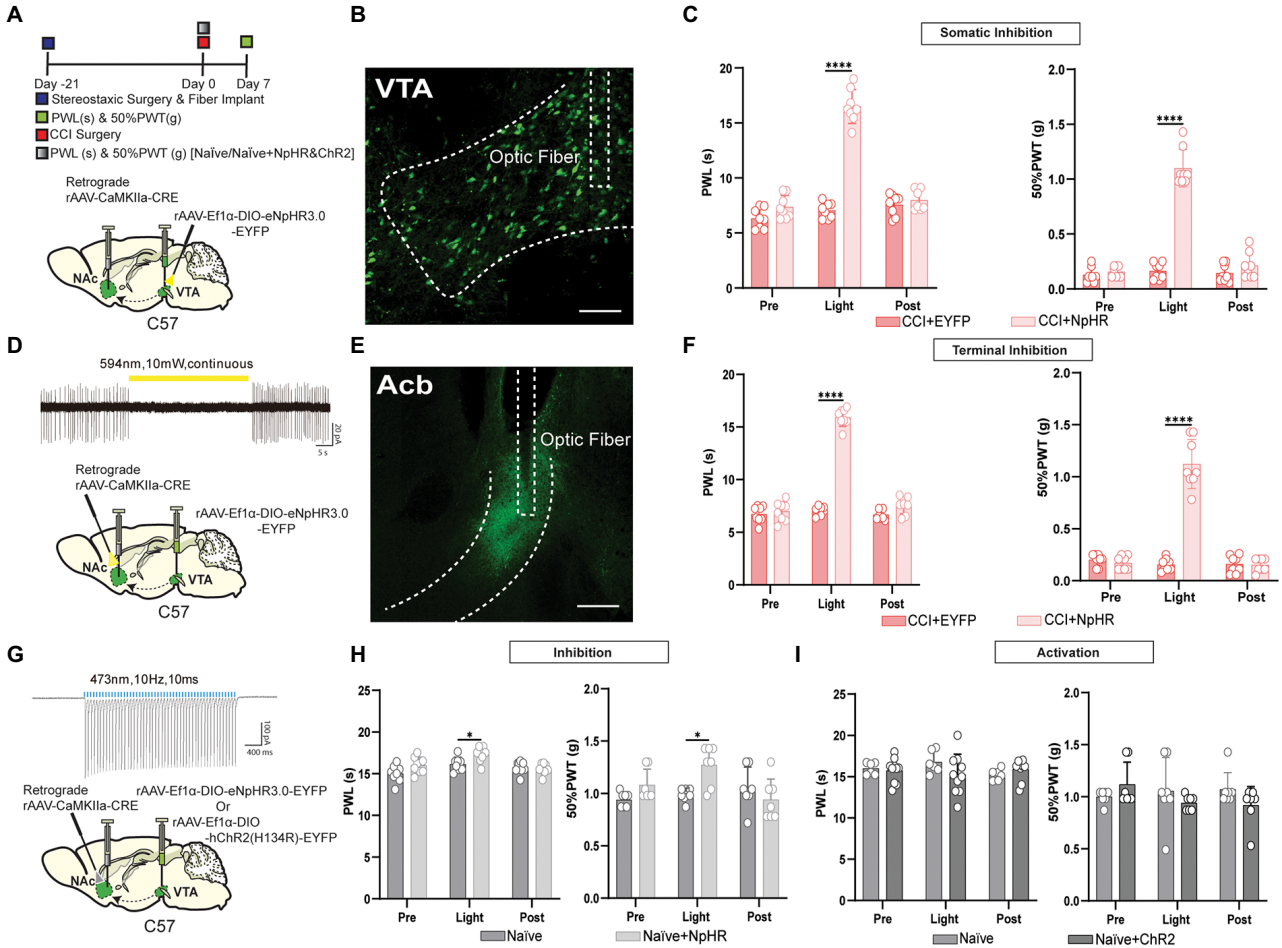
Inhibition of the glutamatergic neurons projected from VTA to NAc has relieved neuropathic pain in wild-type mice. **(A)** Experimental timeline. Schematic illustration depicting viral constructs. For the somatic stimulation, a retrograde CaMKIIa-CRE viral vector was injected in the NAc brain region and DIO-NpHR-EYFP injection with fiber implantation was performed on the VTA, wild-type mice were given 21 days for the expression of the virus before the CCI surgery, PWLs and 50% PWTs of the hind paws were assessed on day 7 after CCI surgery in wild-type mice. **(B)** Representative confocal image for somatic virus expression in VTA of wild-type mice infected by the viral vector of CaMKIIa-CRE and DIO-NpHR-EYFP for labeling glutamatergic projections. Scale bar = 200 μm. **(C)** The quantitative comparison of PWLs and 50% PWTs between the two groups (with and without light stimulation). Statistics show that PWLs of CCI + NpHR group exhibited a significant increase in withdrawal latency during the somatic light stimulation period [Light phase] compared with the CCI + EYFP group (*n* = 8, 8 mice; ^****^p < 0.0001), also 50% PWTs of CCI + NpHR group were also significantly increased during the somatic light stimulation [Light phase] compared with the CCI + EYFP group (*n* = 8, 8 mice; ^***^p < 0.001) of the wild-type mice. **(D)** 594 nm yellow laser stimulation-induced inhibition of firing activity in VTA glutamatergic neuron expressing NpHR with axonal projections to NAc (10 mW, continuous stimulation). For the terminal light stimulation, a retrograde CaMKIIa-CRE viral vector injection with fiber implantation was performed in the NAc brain region, and DIO-NpHR-EYFP was injected into the VTA, wild-type mice were given 21 days for the expression of the virus before the CCI surgery, PWLs and 50% PWTs were assessed on day 7 after CCI surgery. **(E)** Confocal images showing virus expression NAc axon terminals in the brain region of wild-type mice infected by the viral vector of CaMKIIa-CRE and DIO-NpHR-EYFP for labeling glutamatergic projections. Scale bar = 200 μm. **(F)** The quantitative comparison of PWLs and 50% PWTs between the two groups (with and without light stimulation). Statistics showing that CCI + NpHR group exhibited a significant increase in PWLs during the terminal light stimulation [Light phase] compared with the CCI + EYFP group (*n* = 8, 8 mice; ^****^p < 0.0001), 50% PWTs of CCI + NpHR group were also increased during the terminal light stimulation [Light phase] compared with the CCI + EYFP group (*n* = 8, 8 mice; ^***^p < 0.0001) in wild-type mice. **(G)** For the somatic activation of this pathway, a retrograde viral vector CaMKIIa-CRE viral vector were injected in the NAc brain region and DIO-ChR2-EYFP or DIO-NpHR-EYFP was injected in the VTA of naïve mice, 473 nm blue laser stimulation-induced excitation of firing activity in VTA glutamatergic neuron expressing ChR2 with axonal projections to NAc brain region (473 nm, 10 Hz, 10 ms; the validation of EYFP-NpHR viral was described previously for the somatic inhibition) and wild-type mice were given 21 days for the expression of the virus. **(H)** The comparison of PWLs and 50% PWTs between the two groups (with and without light stimulation). Statistics showing that Naïve + NpHR group exhibited a less significant increase in PWLs during the somatic light stimulation [Light phase] compared with the naïve group (*n* = 7, 7 mice; ^*^p < 0.05), 50% PWTs of the Naïve + NpHR group also exhibited a less significant increase by the somatic light stimulation [Light phase] compared with the naïve group (*n* = 7, 7 mice; ^*^p < 0.05). **(I)** During the terminal activation, the quantitative comparison (with and without light stimulation) of PWLs and 50% PWTs between the two groups showed Naïve + NpHR group exhibited no significant difference in PWLs by the somatic stimulation compared with the naïve group (*n* = 7, 7 mice; *p* > 0.05), 50% PWTs of the Naïve+ChR2 group also exhibited no significance with the naïve group (*n* = 7, 7 mice; *p* > 0.05) of the wild-type mice (All the readings were measured during 3 consecutive periods, and all the comparisons were performed by the *two-way ANOVA test* followed by *Bonferroni post-test*).

**Figure 3:**
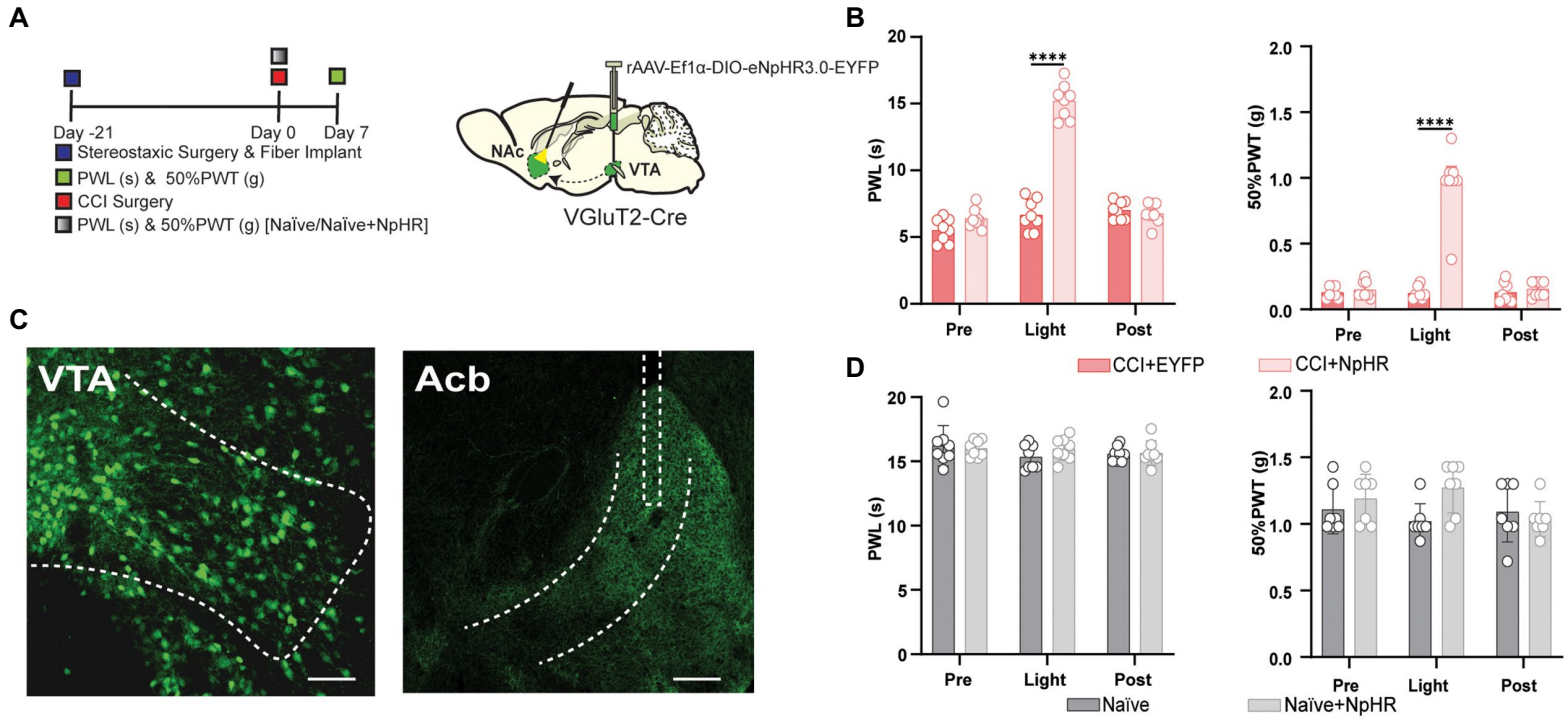
Inhibition of the glutamatergic neurons projected from VTA to NAc has relieved neuropathic pain in VGluT2-Cre mice. **(A)** Experimental timeline. Schematic illustration depicting viral constructs. For the terminal stimulation, DIO-NpHR-EYFP was injected in the VTA, the optical fiber was planted at the NAc in VGluT2-Cre mice, and mice were given 21 days for the expression of the virus before the CCI surgery or before PWLs and 50% PWTs measurement in naïve mice. PWLs and 50% PWTs of the hind paws were assessed on day 7 after CCI surgery in VGluT2-Cre mice. **(B)** The quantitative comparison of PWLs and 50% PWTs between the two groups (with and without light stimulation). Statistics showing that CCI + NpHR group exhibited a significant increase in PWLs during the terminal light stimulation period [Light phase] compared with the CCI + EYFP group (*n* = 8, 8 mice; ^****^p < 0.0001), 50% PWTs of CCI + NpHR group were also significantly increased during the terminal light stimulation [Light phase] compared with the CCI + EYFP group (*n* = 8, 8 mice; ^***^p < 0.0001) in VGluT2-Cre mice. **(C)** Confocal images showing virus expression in the somatic bodies of VTA and terminal projection in NAc brain region of the VGluT2-Cre mice. Scale bar = 200 μm and scale bar = 200 μm. **(D)** The comparison of PWLs and 50% PWTs between the two groups of the naïve mice (with and without light stimulation), Statistics showing Naïve + NpHR group exhibited no significant difference in PWLs and 50% PWTs compared with the Naïve group (*n* = 7, 7 mice; *p* > 0.05) of the VGluT2-Cre mice (All the readings were measured during 3 consecutive periods and the comparisons were performed by the *two-way ANOVA test* followed by *Bonferroni post-test*).

We wanted to investigate the pain-relieving effect of VTA glutamatergic projections to NAc in the CCI-induced neuropathic pain model of wild-type and VGluT2-Cre mice. First, we used two different methods for manipulating the VTA glutamatergic neurons with projections to NAc in wild-type mice. For the somatic inhibition in mice induced by the CCI surgery, A retrograde CaMKIIa-CRE virus was injected into NAc. DIO-EYFP-NpHR injection with optical fibers implantation was performed on the wild-type mice’s VTA brain region. For the control groups, the DIO-EYFP was injected in the VTA without the presence of NpHR (Figure 2A). For terminal inhibition in mice induced by the CCI surgery, a retrograde CaMKIIa-CRE injection with optical fibers implantation was performed onto the NAc brain region, and DIO-EYFP-pHR was injected into VTA of the wild-type mice. For the control groups, the DIO-EYFP was injected in the VTA without the presence of NpHR. For terminal inhibition in wild-type naïve mice, a retrograde CaMKIIa-CRE injection with optical fibers implantation was performed onto the NAc brain region, and DIO-EYFP-NpHR was injected into VTA of the wild-type mice, For the control groups, the DIO-EYFP was injected in the VTA without the presence of NpHR. For terminal activation in wild-type naïve mice, a retrograde CaMKIIa-CRE injection with optical fibers implantation was performed onto the NAc brain region, and DIO-EYFP-ChR2 was injected into VTA of the wild-type mice, For the control groups, the DIO-EYFP was injected in the VTA without the presence of ChR2. Besides, electrophysiological stimulation was also done to verify the effective manipulation of the viral vector (Figures 2D,G). Confocal imaging was performed for the viral expression in VTA and NAc (Figures 2B,E). We noted that the mice had a pain-reducing effect through optogenetic inhibition of VTA glutamatergic projections to NAc in CCI mice infected with DIO-EYFP-NpHR compared to control groups (Figures 2C,F). In addition, this inhibitory effect of the VTA glutamatergic projections to NAc was bidirectional, as the somatic and terminal stimulation both induce the same pain-relieving effect of the neuropathic pain. The naïve mice with artificial inhibition of glutamatergic neurons projected from VTA to NAc showed less elevation in the pain intensity. Surprisingly, the activation of these neurons through DIO-ChR2-EYFP could not induce any change in the thermal and mechanical pain thresholds (Figures 2H,I). Although multiple types of VTA VGluT2 neurons may target the same output structure, it has so far been shown that VGluT2-only neurons project to the nucleus accumbens (Root et al., 2018). To further reveal this, we injected DIO-EYFP-NpHR in VTA, and the optical fibers were planted in the NAc brain region of VGluT2-Cre mice induced by the CCI surgery (Figure 3A). Similar to the results in wild-type mice, we found that optogenetic inhibition of specific VTA glutamate projections to NAc suppressed the thermal and mechanical pain threshold in CCI mice (Figures 3B,C). Besides, artificial activation through the DIO-ChR2 viral vector or inhibition through DIO-NpHR of these VTA-NAc glutamate projections did not affect the pain behavior in the naïve VGluT2-Cre mice (Figure 3D).

### The photoinhibition of glutamatergic neurons projected from VTA to NAc suppressed CFA injection-induced acute and chronic inflammatory pain

**Figure 4:**
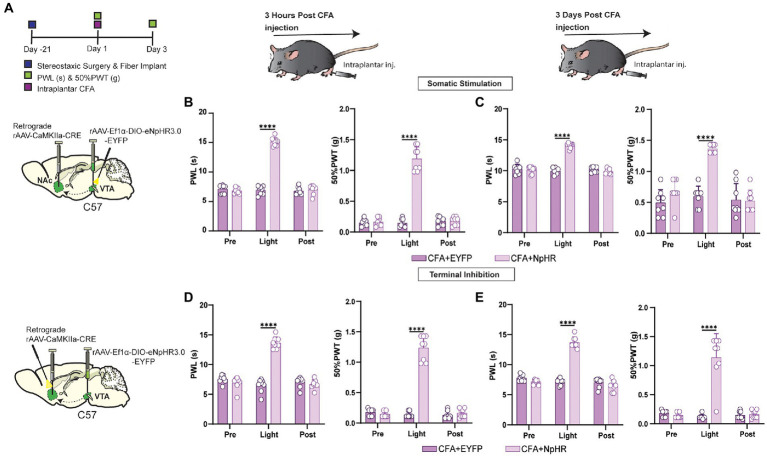
Inhibition of this pathway can affect both short-term and long-term inflammatory pain in wild-type mice. **(A)** Experimental timeline. Schematic illustration depicting viral constructs. For the somatic stimulation, a retrograde CaMKIIa-CRE viral vector was injected in the NAc brain region, and DIO-NpHR-EYFP injection with fiber implantation was performed on the VTA, and wild-type mice were given 21 days for the expression of the virus before the ip.l. CFA injection. 50% PWTs and PWLs of the hind paws were assessed at 3 h and day 3 after ip.l. CFA injection in wild-type mice. **(B)** The quantitative comparison of PWLs and 50% PWTs between the two groups (with and without light stimulation). Statistics show that PWLs of CFA + NpHR group exhibited a significant increase in withdrawal latency by the somatic light stimulation [Light phase] compared to the CFA + EYFP group (*n* = 8, 8 mice ^****^p < 0.0001), also 50% PWTs of CFA + NpHR group were significantly increased during the somatic light stimulation [Light phase] compared with the CFA + EYFP group (*n* = 8, 8 mice; ^***^p < 0.0001) of the wild-type mice after 3 h of ip.l. CFA injection. **(C)** The quantitative comparison of PWLs and 50% PWTs between the two groups (with and without light stimulation). Statistics showing that PWLs of the CFA + NpHR group exhibited a significant increase in withdrawal latency during the somatic light stimulation [Light phase] compared with the CFA + EYFP group (*n* = 8, 8 mice ^****^p < 0.0001). Also, 50% PWTs of CFA + NpHR group were significantly increased during the somatic light stimulation [Light phase] compared with the CFA + EYFP group (n = 8, 8 mice; ***p < 0.0001) of the wild-type mice after 3 days of ip.l. CFA injection. **(D)** For the terminal stimulation, a retrograde CaMKIIa-CRE viral vector injection with fiber implantation was performed on the NAc brain region, and DIO-NpHR-EYFP was injected in the VTA. PWLs and 50% PWTs of the hind paws were assessed at 3 h and day 3 after ip.l. CFA injection in wild-type mice. The quantitative comparison (with and without light stimulation) of PWLs and 50% PWTs between the two groups. Statistics showing that PWLs of CFA + NpHR group exhibited a significant increase in withdrawal latency during the terminal light stimulation [Light phase] period compared with the CFA + EYFP group (*n* = 8, 8 mice; ^****^p < 0.0001). Also, 50% PWTs of CFA + NpHR group were significantly increased during the terminal light stimulation [Light phase] compared with the CFA + EYFP group (*n* = 8, 8 mice; ^****^p < 0.0001) of the wild-type mice after 3 h of ip.l. CFA injection. **(E)** Statistics showing that PWLs of CFA + NpHR group exhibited a significant increase in withdrawal latency during the terminal light stimulation period [Light phase] compared with the CFA + EYFP group (n = 8, 8 mice; ^****^p < 0.0001). Also, 50% PWTs of CFA + NpHR group were significantly increased during the terminal light stimulation period [Light phase] compared with the CFA + EYFP group (n = 8, 8 mice; ^****^p < 0.0001) of the wild-type mice after 3 days of ip.l. CFA injection (All the readings were measured during 3 consecutive periods, and the comparisons were performed by the *two-way ANOVA test* followed by *Bonferroni post-test*).

**Figure 5:**
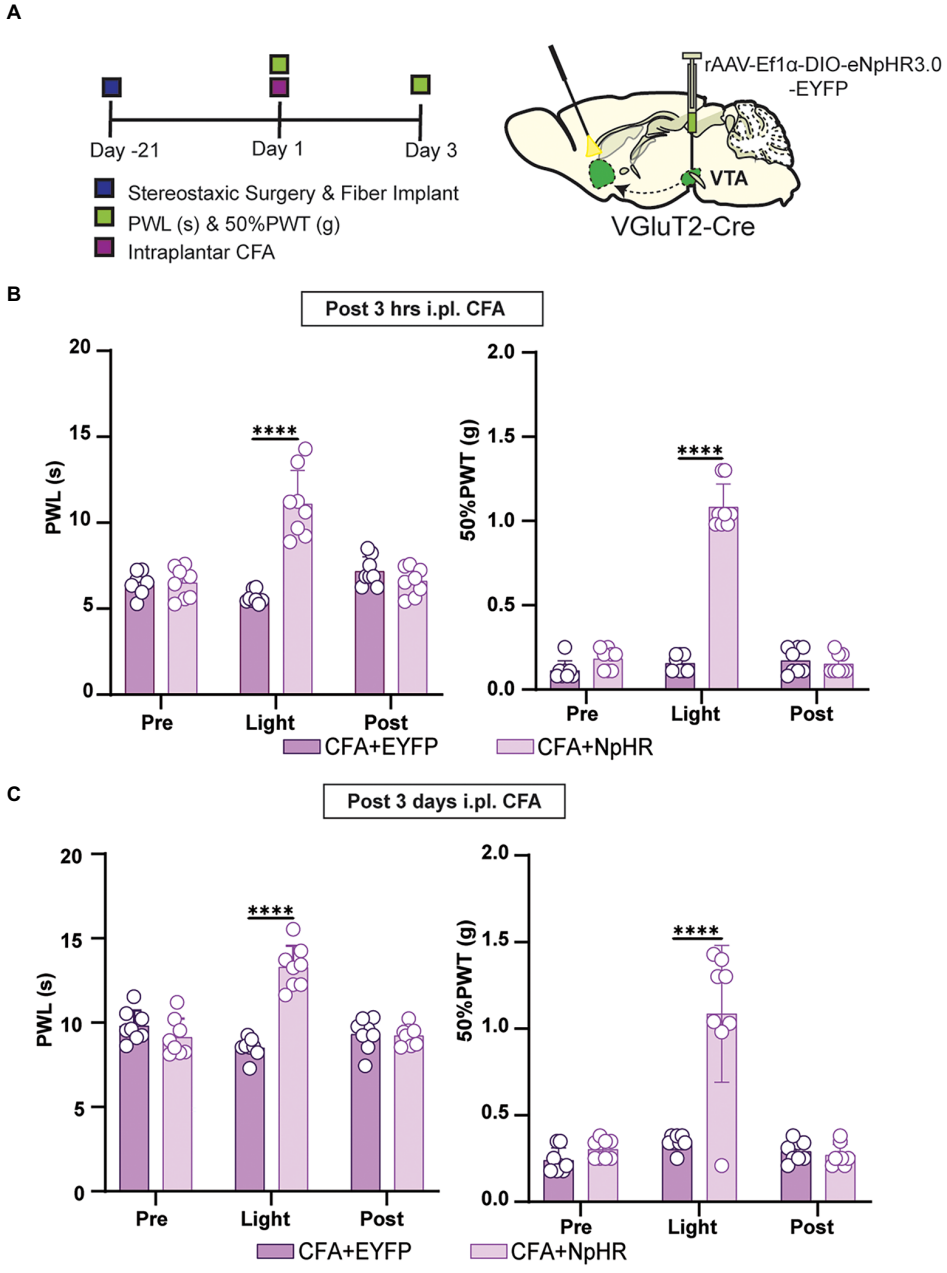
Inhibition of this pathway can affect both short-term and long-term inflammatory pain in VGluT2-Cre mice. **(A)** Experimental timeline. Schematic illustration depicting viral constructs. For stimulation, DIO-NpHR-EYFP was injected in the VTA, the optical fiber was planted at the NAc in VGluT2-Cre mice, mice were given 21 days for the expression of the virus before the ip.l. CFA injection. PWLs and 50% PWTs of the hind paws were assessed at 3 h and day 3 after ip.l. CFA injection in VGluT2-Cre mice. **(B)** The quantitative comparison of PWLs and 50% PWTs between the two groups (with and without light stimulation). Statistics showing that CFA + NpHR group exhibited a significant increase in PWLs during the terminal light stimulation [Light phase] compared with the CFA + EYFP group (*n* = 8, 8 mice; ^****^p < 0.0001), 50% PWTs of CFA + NpHR group were also increased by the terminal light stimulation [Light phase] compared with the CFA + EYFP group (*n* = 8, 8 mice; ^***^p < 0.0001) in VGluT2-Cre mice after 3 h of ip.l. CFA injection. **(C)** The quantitative comparison of PWLs and 50% PWTs between the two groups (with and without light stimulation). Statistics showing that CFA + NpHR group exhibited a significant increase in PWLs by the terminal light stimulation period [Light phase] compared with the CFA + EYFP group (*n* = 8, 8 mice; ^****^p < 0.0001), 50% PWTs of CFA + NpHR group were also increased during the terminal light stimulation phase [Light phase] compared with the CFA + EYFP group (*n* = 8, 8 mice; ^****^p < 0.0001) in VGluT2-Cre mice after 3 days of ip.l. CFA injection (All the readings were measured during 3 consecutive periods, and the comparisons were performed by the *two-way ANOVA test* followed by *Bonferroni post-test*).

Is the pain-reducing effect of suppressing VTA-NAc glutamatergic projection sustained in acute and chronic inflammatory pain? To answer this question, the short-term and long-term CFA injection-induced pain models were prepared as acute and chronic stages of pain reported previously (Ling et al., 2020). For the somatic stimulation in wild-type mice induced by the intraplantar hind paw injection of CFA, a retrograde CaMKIIa-CRE virus was injected into NAc, and DIO-EYFP-NpHR was injected with optical fiber implantation on VTA. For terminal stimulation in wild-type mice induced by the intraplantar hind paw injection of CFA, a retrograde CaMKIIa-CRE was injected with fiber implantation onto NAc, and DIO-EYFP-NpHR was injected into VTA (Figure 4A). The data showed that inhibiting VTA-NAc glutamatergic projection shows a decrease in pain intensity at 3 h and at 3 days after CFA injection (Figures 4B–E). For further confirmation, the VGluT2-Cre mice induced by intraplantar hind paw injection of CFA were used. We injected DIO-EYFP-NpHR in VTA, and the optical fibers were planted on NAc in VGluT2-Cre mice (Figure 5A). Inhibiting VTA-NAc glutamatergic projection among VGluT2-Cre mice also showed a decrease in pain intensity at 3 h and at 3 days after CFA injection (Figures 5B,C). These data suggested that VTA glutamatergic projections to NAc are equally effective in both acute and chronic inflammatory pain states. In contrast, the optogenetic inhibition by DIO-EYFP-NpHR or activation by DIO-EYFP-ChR2 of VTA glutamatergic projections to NAc did not effect the pain state in VGluT2-Cre mice.

### The inhibition of glutamatergic neurons projected from VTA to NAc released chronic pain-related anxiety

**Figure 6:**
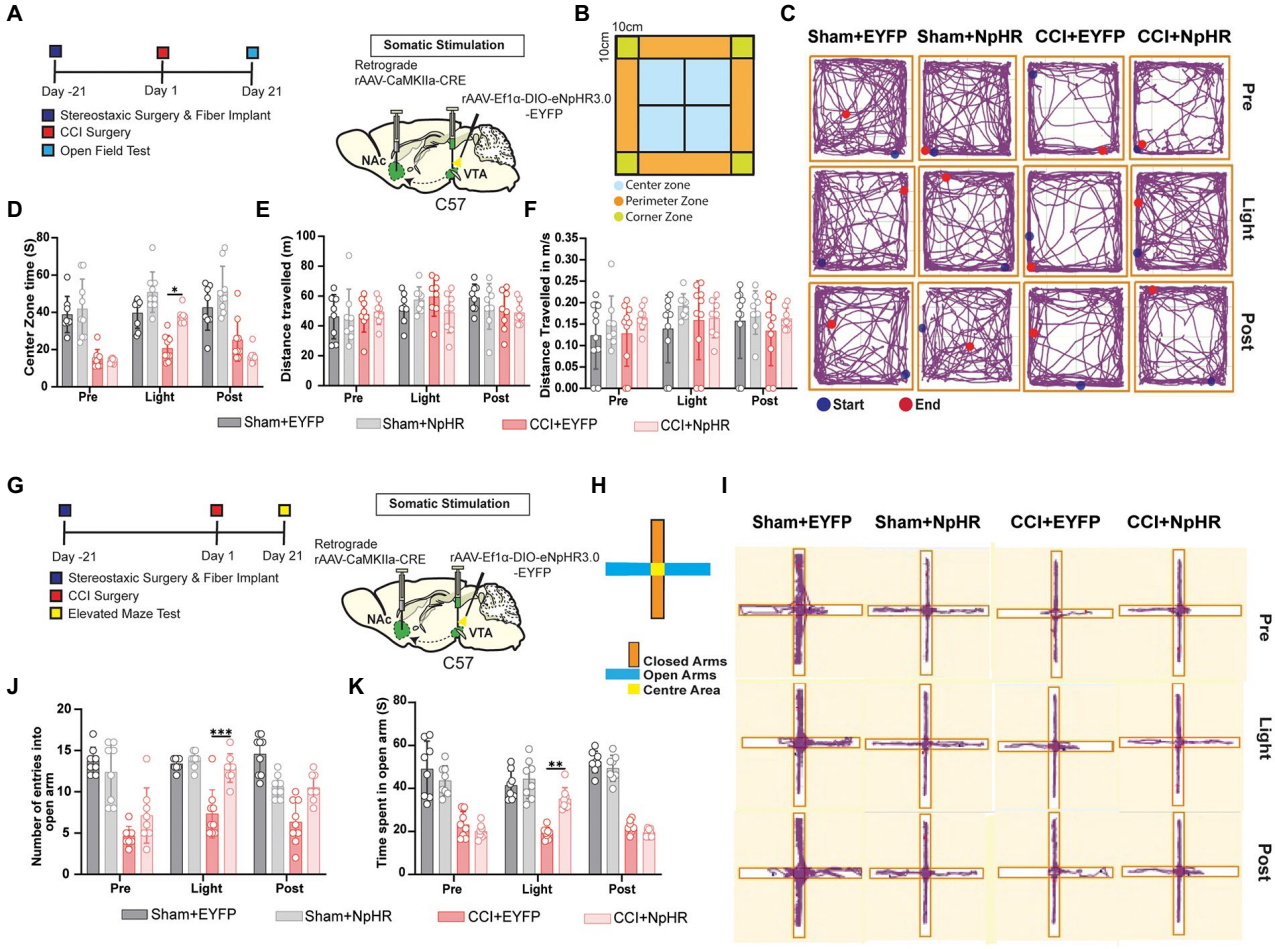
Inhibition of glutamatergic neurons projected from VTA to NAc decreases anxiety and improves the exploratory behavior of mice. **(A)** Experimental timeline. Schematic illustration depicting viral constructs. For the somatic stimulation, a retrograde viral vector CaMKIIa-CRE viral vector was injected in the NAc brain region and DIO-NpHR-EYFP was injected in the VTA, and the optical fibers were also planted at the VTA, mice were given 21 days for the expression of the virus before the CCI or sham surgery. OFT was performed on day 21 after CCI surgery was established by the ligation of sciatic nerve and sham was produced with only an incision but without the ligation of sciatic nerve in wild-type mice. **(B)** Illustrated design of the field area for the OFT behavioral tests. **(C)** Tracing image of Sham + EYFP, Sham + NpHR, CCI + EYFP, and CCI + NpHR groups defining the roaming field of mice in different time periods. **(D)** The quantitative comparison regarding the time spent in the center zone by the groups of Sham + EYFP, Sham + NpHR, CCI + EYFP and CCI + NpHR (with and without light stimulation). Statistics showing that there is a significant difference regarding time duration spent in the center zone of CCI + NpHR group compared with the CCI + EYFP group (*n* = 8, 8, 8, 8 mice; ^*^p < 0.05) but no significant difference compared to Sham + EYFP and Sham + NpHR (*n* = 8, 8, 8, 8 mice; *p* > 0.05) during the light stimulation period [Light phase]. **(E)** The quantitative comparison regarding distance traveled by the groups of Sham + EYFP, Sham +N pHR, CCI + EYFP, and CCI + NpHR. Statistics showing that all the groups showed no statistical difference in any time period. (*n* = 8, 8, 8, 8 mice; *p* > 0.05). **(F)** The quantitative comparison regarding speeds of Sham + EYFP, Sham + NpHR, CCI + EYFP, and CCI + NpHR. Statistics showing that all the groups showed no statistical difference in any time period. (*n* = 8, 8, 8, 8 mice; *p* > 0.05). **(G)** Experimental timeline. Schematic illustration depicting viral constructs. For the somatic stimulation a retrograde viral vector CaMKIIa-CRE viral vector was injected in the NAc brain region and DIO-NpHR-EYFP was injected in the VTA, the optical fiber was also implanted at the VTA, mice were given 21 days for the expression of the virus before the CCI or sham surgery. EMT was performed on day 21 after CCI surgery in wild-type mice. The ligation of sciatic nerve established the CCI pain model. Sham was produced with only an incision but without sciatic nerve ligation. **(H)** Illustrated design regarding the arms of elevated maze. **(I)** Tracing image of Sham + EYFP, Sham + NpHR, CCI + EYFP, and CCI + NpHR groups defining the roaming field of mice in different time periods. **(J)** The quantitative comparison regarding the number of entries in the open arms of elevated maze by the mice of the groups of Sham + EYFP, Sham + NpHR, CCI + EYFP, and CCI + NpHR (with and without light stimulation). Statistics show that CCI + EYFP group exhibited a significant decrease in the number of entries in open area compared with CCI + NpHR group (*n* = 8, 8, 8, 8 mice; ^***^p < 0.001), but CCI + NpHR had no significant difference compared to Sham + EYFP, Sham + NpHR during the somatic light stimulation [Light phase] (*n* = 8, 8, 8, 8 mice; *p* > 0.05). **(K)** Statistics showing that CCI + EYFP group exhibited a significant decrease in the time spent in open area compared with CCI + NpHR group (*n* = 8, 8, 8, 8 mice; ^**^p < 0.01), but CCI + NpHR had no significant difference compared to Sham + EYFP, and Sham + NpHR during the somatic light stimulation [Light phase] (*n* = 8, 8, 8, 8 mice; *p* > 0.05; all comparisons were done by *two-way ANOVA test* followed by *Bonferroni post-tests*).

Previously researchers reported that animal subjects suffering from chronic pain could develop anxiety-like behavior (Li et al., 2014; Zhou et al., 2020; Liu et al., 2021). The open field and elevated maze tests (Murasawa et al., 2020) were used for testing anxiety 21 days after CCI or sham surgery. For the somatic stimulation in wild-type mice, a retrograde CaMKIIa-CRE virus was injected into NAc, and DIO-EYFP-NpHR injection with fiber implantation was placed on the VTA brain region, (Figures 6A,B). Compared with the control subject, CCI surgery resulted in decreased latency to enter the center area after being placed into the apparatus, and the photoinhibition of VTA-NAc glutamatergic pathway improved the exploratory behavior of CCI mice compared to its control counterparts and sham groups (Figures 6C,D). Besides this, the locomotion rate has not been influenced among all the groups (Figures 6E,F). Secondly, we performed the elevated maze test (EMT), 21 days after CCI or sham surgery. Again, for the somatic stimulation in wild-type mice, a retrograde CaMKIIa-CRE virus was injected into NAc, and DIO-EYFP-NpHR was injected with fiber implantation placed on VTA of the wild-type mice (Figures 6G,H). The EMT showed that CCI surgery resulted in a decreased duration of time spent and the number of entries in the open arms; the optogenetic inhibition of VTA-NAc glutamatergic pathway rescued this decrease among CCI-NpHR group significantly (Figures 6I–K). These data suggested that glutamatergic neurons projected from VTA to NAc contributed to pain-related anxiety behaviors.

### The inhibition of glutamatergic neurons projected from VTA to NAc released chronic pain-related depression

**Figure 7:**
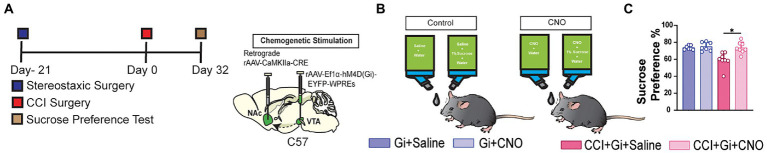
Inhibition of glutamatergic neurons projected from VTA to NAc can reduce the pain-induced decrease in sucrose preference, and this behavior. **(A)** Experimental timeline. Schematic illustration depicting viral constructs. For the chemogenetic stimulation a retrograde CaMKIIa-CRE viral vector was injected in the NAc brain region, and DIO-hM4D(Gi)-EYFP was injected in the VTA, mice were given 21 days for the expression of the virus before the CCI surgery, the sucrose test was performed at the 32 days post-surgery in the CCI-induced mice along with the mice without CCI surgery. **(B)** The illustration of the oral route of CNO infusion, CNO was mixed with water and 1% sucrose solution and the control group fluids were prepared without the presence of CNO drug. **(C)** Statistics data showing that CCI + Gi + Saline exhibited a significant decrease compared with CCI + Gi + CNO, Gi + Saline and Gi + CNO (*n* = 7, 7, 8, 8 mice; ^*^p < 0.05) during the testing of sucrose preference of 24 h (All the data were measured by *one-way ANOVA* followed by the *Dunnett’s test*).

**Figure 8:**
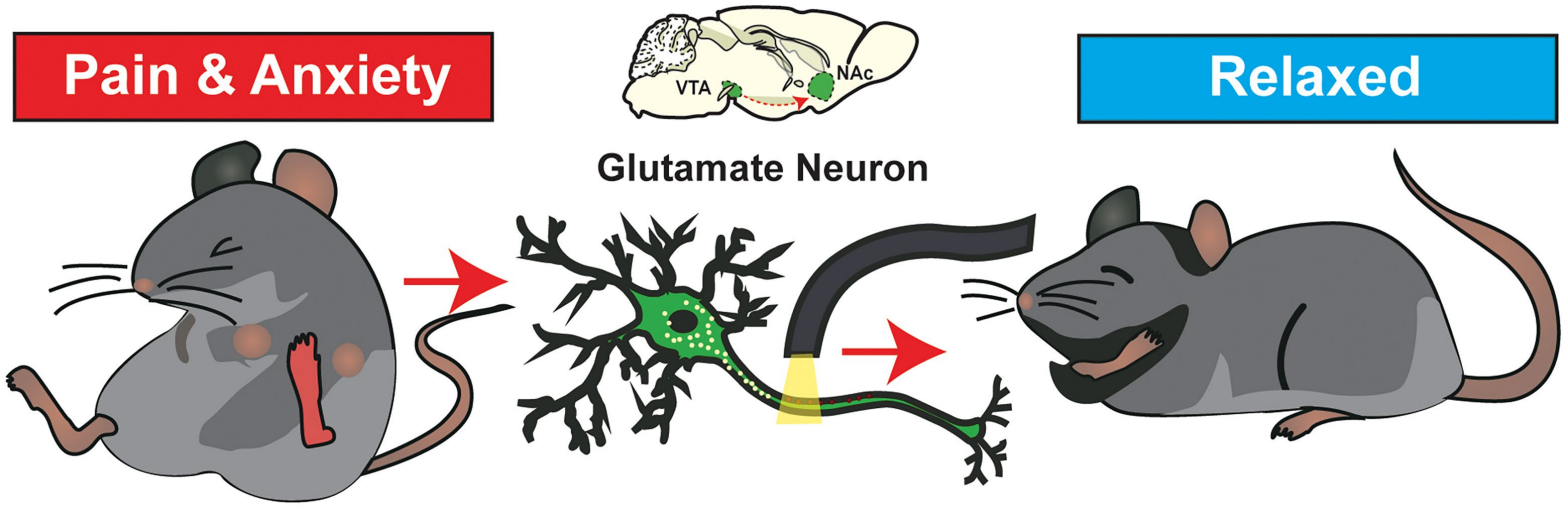
Schematic of theoretical pathway involvement in pain and pain-related behavior. Illustrated figure shows the VTA glutamate afferents to the NAc. These projections played a role in causing unrest and anxiety-like psychological mood change among the subjected mice suffering from pain stimuli. When these projections are silenced, it can relieve the mice from pain and pain-induced anxiety.

It was reported that pain-induced depression evolves after pain has lasted at least 4–5 weeks (Llorca-Torralba et al., 2022), and the sucrose preference test is known for measuring stress-induced anhedonia in mice (Li et al., 2017; Liu et al., 2018). The intraperitoneal injection of CNO could create a bias in the experiment, so we utilized the infusion of CNO by mixing it with the desired solution as described previously (Zhan et al., 2019). We injected retrograde CaMKIIa-CRE into NAc and EYFP-hM4D (Gi) into the VTA of the wild-type mice (Figures 7A,B). The sucrose preference was recorded within 24 h among mice after 32 days of CCI surgery, and at the same time for the mice without the CCI surgery. Compared to control group, CCI-induced pain mice resulted in a reluctance and a significant decline in the preference for drinking the 1% sucrose water. The chemogenetical inhibition of the glutamatergic neurons projected from VTA to NAc could significantly improve the sucrose preference among mice suffering from CCI-induced pain compared to its control counterparts (Figure 7C). These data suggested that glutamatergic neurons projected from VTA to NAc contributed to pain-related depression behaviors too (Figure 8).

## Discussion

Pain triggers maladaptive changes within the mesolimbic system (Massaly et al., 2016), leading to negative affective states such as anxiety and other related behavioral changes (Sieberg et al., 2018). The DA neurons in the VTA are considered the famous therapeutic target for treating reward-related behaviors, such as drug addictions and mood disorders, due to their crucial roles in directing reward-related responses. VTA GABA neurons have also been found to regulate reward consumption, depression, stress, and sleep by altering DA release from adjacent DA neurons, suggesting that the function of VTA GABA neurons is partially dependent on DA release. DA neurons are nominated explicitly for addiction (Pascoli et al., 2015), and relatively fewer researchers have focused on VTA glutamate signaling due to their intermittent existence in the VTA. However, recent studies suggested that VTA glutamate neurons regulate reward reinforcement, aversive behaviors, wakefulness, and defensive behaviors. However, its role in pain and related behavioral changes needs more detailed investigations.

In our study, we observed that glutamatergic neurons of VTA had efferent inputs to the NAc, which was constant in the previous study (Qi et al., 2016), and these VTA glutamatergic projections to NAc could be triggered for an increased firing rate and neuronal activity by inducing pathological pain of chronic constrictive injury providing a well-defined proposal that these glutamatergic projections from VTA to NAc also contributes to pain.

Acute activation of VTA glutamatergic terminals in the LHb, VP, and NAc induces self-stimulation and aversion through glutamate-mediated action (Qi et al., 2016), and the local VGluT2 levels in the nucleus accumbens are reported to remain unaltered in the pain state (Yamaguchi et al., 2007). In contrast, another report suggested that VGluT2 expression is essential in pain behavior (Scherrer et al., 2010). We found that activation of both CaMKII coupled and VGluT2 projections of VTA had no-significant changes in pain behavior. Still, in comparison, the inhibition of these projections can induce pain relief effects for both acute and chronic pain. In our idea, the VGluT2 inputs from VTA produce an inhibitory response to pain behavior by creating a rewarding aspect of relief in NAc (Harris and Peng, 2020).

People with chronic pain have three times the average risk of developing psychiatric symptoms like anxiety disorders and depression (Woo, 2010; Wilmer et al., 2021). Measurements of anxiety with chronic pain also show a strong association with depression. Neuropathic pain, initiated by constriction of the sciatic nerve, can induce anxiety-like behavior in mice, and the mice could develop a fear (Kamonseki et al., 2021). VTA-glutamate neuronal activity response to innate threatening stimuli (Barbano et al., 2020) and inhibitory response of NAc local glutamate receptors can prevent depressive symptoms. Moreover, neuropathic pain can induce anxiety, depression, and pain-related fear (Bagot et al., 2015; Xu et al., 2020). Our finding also proposed that chronic pathological pain can cause anxiety-like fear behavior in mice which can be reduced by inhibiting VTA glutamate projections to NAc. We can propose that this can be due to the way VTA glutamate neurons regulates the activity of D1 and D2 receptors in NAc (Sato et al., 2022) and inhibition of the D2 receptor alone can reduce the pain behavior among mice (Supplementary Figure 1). Another proposal could be the interneurons signaling in dopamine/glutamate interaction at different sites for behavioral responses (Richard and Berridge, 2011; Xiao et al., 2021). However, it is still unclear how the VTA-NAc-specific molecular mechanisms modulate pain perception. Future studies could use the projection- and cell-type-specific gene analysis techniques and live single-cell sequencing techniques for further exploration.

Our study has some limitations. Firstly, this only defines the role of VTA glutamatergic inputs to the NAc brain region. We have planned future studies to compare different brain regions and types of neurons like DA and GABA for a comparative outcome. In our study, we used CCI pain as the primary pain model for all the anxiety and depressive changes that could cause different approaches in molecular-level mechanisms of CFA. We presumed that the behavioral response remains the same as reported in a study done previously (Zhou et al., 2021). Third, although D2 receptors positive neurons in the NAc contributed to the pain behavior, we did not examine the directed role of the glutamatergic inputs from VTA on these subtype neurons in the pain and pain-related behaviors, which needs to be further investigated.

## Conclusion

We can summarize that VTA glutamatergic neurons with projections to NAc are as critical as the role of dopamine neurons in pain and pain-induced anxiety. We proposed that this inhibitory pathway could reduce the dopamine-only mediated side effects observed in recreational and clinical settings. The study is one of the first to discover the inhibition role of the VTA-glutamatergic pathway in pain mechanism and nociceptive induce anxiety. It may enlighten further studies focusing on understanding the multiple functions of VTA glutamatergic neurons.

## Data availability statement

The original contributions presented in the study are included in the article/Supplementary material, further inquiries can be directed to the corresponding author.

## Author contributions

MA and J-LC designed the study, critically reviewed the manuscript, approved the final version, and was accountable for the work. MA and H-QY designed the study, analyzed the data, interpreted the data, prepared the manuscript, critically reviewed the manuscript, approved the final version, were accountable for the work, and assisted with the sample testing and data analysis. MA, H-QY, W-NZ, X-BL, ZX, X-LY, and J-LC conducted the study and critically reviewed the data. All authors contributed to the article and approved the submitted version.

## Funding

This work was supported by the National Key R&D Program of China-the Sci-Tech Innovation 2030 Major Project (2021ZD0203100 to J-LC), the National Natural Science Foundation of China (81720108013, 82130033, and 82293641 to J-LC), the Natural Science Foundation of the Jiangsu Higher Education Institutions of China (19KJB320023 to X-LY), the Innovation and Entrepreneurship Program of Xuzhou Medical University (2021CXFUZX002 to X-LY), and Innovation Training Program for College Students in Jiangsu Province (201910313023Z).

## Conflict of interest

The authors declare that the research was conducted in the absence of any commercial or financial relationships that could be construed as a potential conflict of interest.

## Publisher’s note

All claims expressed in this article are solely those of the authors and do not necessarily represent those of their affiliated organizations, or those of the publisher, the editors and the reviewers. Any product that may be evaluated in this article, or claim that may be made by its manufacturer, is not guaranteed or endorsed by the publisher.

## Supplementary material

SUPPLEMENTARY FIGURE 1 Optogenetical inhibition of NAc D2 MSNs suppressed pain behavior. **(a)** Experimental timeline. Schematic illustration depicting viral constructs, mice were given 21 days for the expression of the virus. For the stimulation, DIO-NpHR-EYFP was injected in the NAc and optical fiber was planted at the NAc in D1-Cre/D2-Cre mice. **(b)** PWLs (n = 8, 8) and 50% PWTs (n = 8, 8) were measured during three consecutive periods with or without 594 nm laser stimulation in D1-Cre mice exhibited no significant difference (n = 8, 8 *p* > 0.05). **(c)** The quantitative comparison of PWLs and 50% PWTs between the two groups. Statistics showing that PWLs (n = 8, 8 *****p* < 0.0001) and PWTs (n = 8, 8 *****p* < 0.0001) exhibited a significant decrease in PWLs and 50% PWTs measured during 3 consecutive periods during laser stimulation [Light phase] in D2-Cre mice. All the readings were measured during 3 consecutive periods and all the comparisons were performed by *two-way ANOVA test* followed by *Bonferroni post-test*.

SUPPLEMENTARY FIGURE 2 Neuronal Activity of Glutamate neurons was intact in the chronic pain stage. **(a)** Experimental timeline. For the immunohistological expression. **(b)** The DIO-EYFP viral vector was injected in the VTA brain region and mice were given 21 days for the expression of the virus before the CCI surgery and immunohistological and confocal imaging was performed 30 days after CCI surgery. **(c)** Confocal image of VTA somatic expression of DIO-EYFP in VGluT2-Cre mice (Scale bar = 200 μm).
