# Supplementary figures and images for: VTA-NAc glutaminergic projection involves in the regulation of pain and pain-related anxiety
Source: Front Mol Neurosci. 2022 Dec 7;15:1083671. doi: 10.3389/fnmol.2022.1083671 (PMC9798784; doi:10.3389/fnmol.2022.1083671)

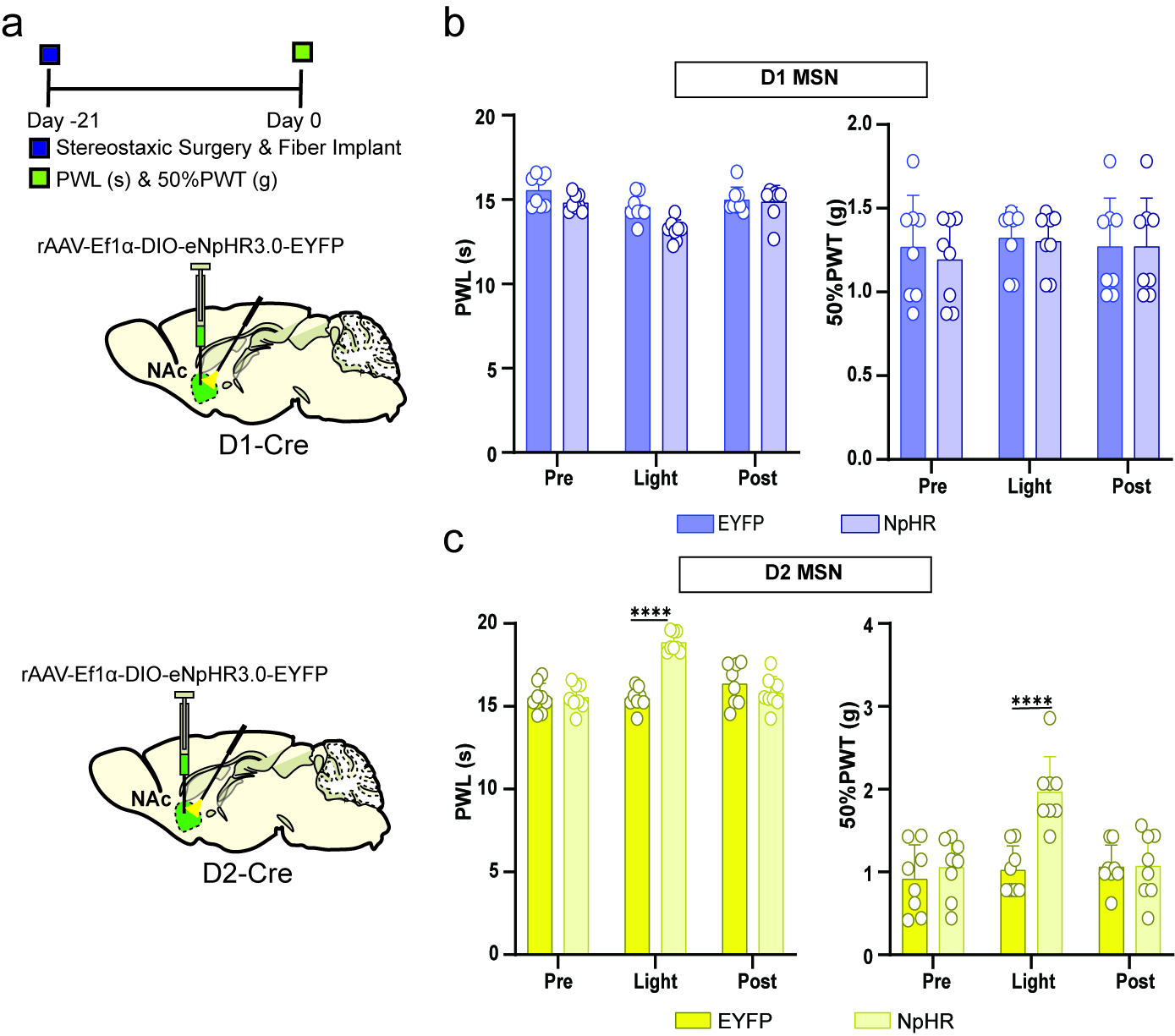

Supplement: SUPPLEMENTARY FIGURE 1 — Optogenetical inhibition of NAc D2 MSNs suppressed pain behavior. (a) Experimental timeline. Schematic illustration depicting viral constructs, mice were given 21 days for the expression of the virus. For the stimulation, DIO-NpHR-EYFP was injected in the NAc and optical fiber was planted at the NAc in D1-Cre/D2-Cre mice. (b) PWLs (n = 8, 8) and 50% PWTs (n = 8, 8) were measured during three consecutive periods with or without 594 nm laser stimulation in D1-Cre mice exhibited no significant difference (n = 8, 8 p > 0.05). (c) The quantitative comparison of PWLs and 50% PWTs between the two groups. Statistics showing that PWLs (n = 8, 8 ****p < 0.0001) and PWTs (n = 8, 8 ****p < 0.0001) exhibited a significant decrease in PWLs and 50% PWTs measured during 3 consecutive periods during laser stimulation [Light phase] in D2-Cre mice. All the readings were measured during 3 consecutive periods and all the comparisons were performed by two-way ANOVA test followed by Bonferroni post-test. [file Image_1.TIF]

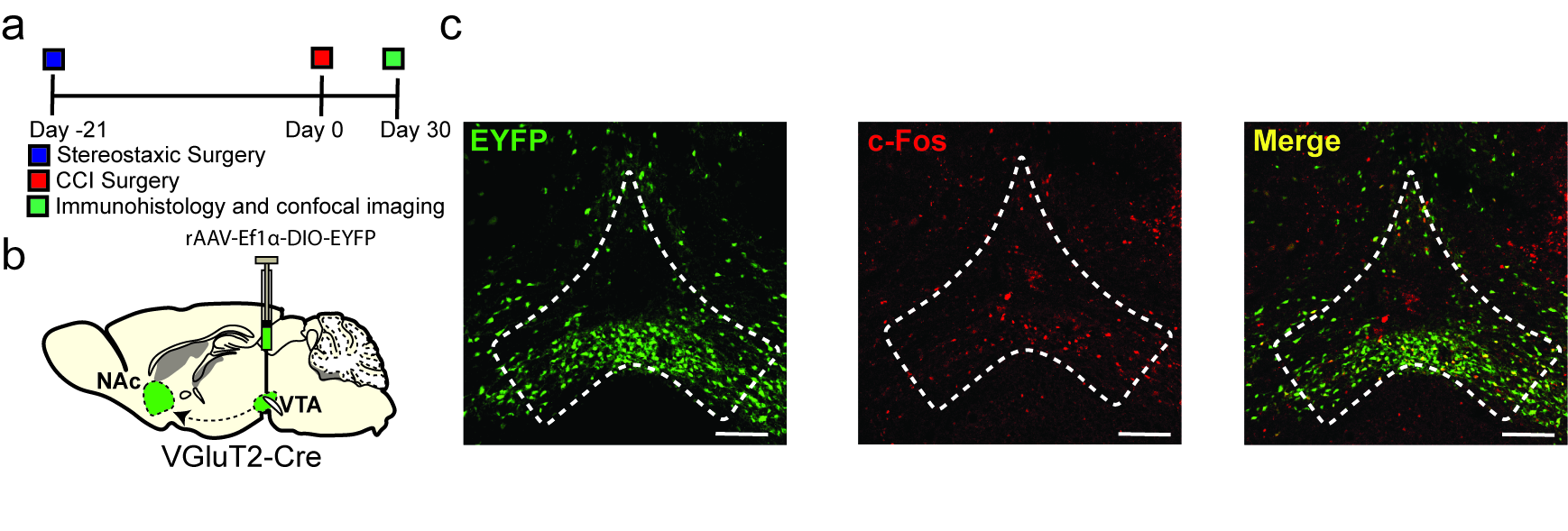

Supplement: SUPPLEMENTARY FIGURE 2 — Neuronal Activity of Glutamate neurons was intact in the chronic pain stage. (a) Experimental timeline. For the immunohistological expression. (b) The DIO-EYFP viral vector was injected in the VTA brain region and mice were given 21 days for the expression of the virus before the CCI surgery and immunohistological and confocal imaging was performed 30 days after CCI surgery. (c) Confocal image of VTA somatic expression of DIO-EYFP in VGluT2-Cre mice (Scale bar = 200 μm). [file Image_2.TIF]
